# Evaluation of dietary composition between hemoglobin categories, total body iron content and adherence to multi-micronutrients in preschooler residents of the highlands of Puno, Peru

**DOI:** 10.1186/s40795-024-00837-x

**Published:** 2024-02-12

**Authors:** Benita Maritza Choque-Quispe, Cinthya Vásquez-Velásquez, Gustavo F. Gonzales

**Affiliations:** 1 Facultad de Ciencias de la Salud, Universidad Nacional del Altiplano, Puno, Peru; 2 Laboratorio de Endocrinología y Reproducción (Laboratorios de Investigación y Desarrollo), Departamento de Ciencias Biológicas y Fisiológicas, Facultad de Ciencias e Ingeniería, Universidad Peruana Cayetano Heredia, Lima, Peru; 3 Instituto de Investigaciones de la Altura, Universidad Peruana Cayetano Heredia, Lima, Peru

**Keywords:** Preschoolers, Anemia, Iron, Diet, High Altitude

## Abstract

**Background:**

The anemia prevalence is higher in highlands populations. It is assumed that iron deficiency anemia (IDA) in children is mainly due to low dietary intake. However, other suggest that high prevalence of anemia is due to an inappropriate hemoglobin (Hb) adjustment for altitude.

**Materials and methods:**

Cross-sectional study conducted in 338 preschoolers (PSC) from Puno-Peru. Hb was measured in whole blood, and ferritin, Soluble transferrin receptor, and Interleukin 6 in serum.The dietary iron intake was assessed by 24-h dietary recall, using NutriCap Software. Hb concentration was assessed as adjusted or unadjusted for altitude.

**Results:**

With unadjusted Hb, the anemia prevalence was 4.7%, whereas after Hb correction, the prevalence raised-up to 65.6% (*p* < 0.001). Reciprocally, erythrocytosis proportion decreased from 20.35 to 0.30% (*p* < 0.001). Total Body Iron (TBI) showed that 7.44% had ID and 0.32% had IDA. PSC with normal unadjusted Hb levels have more protein and micronutrients intake than anemic ones. PSC with erythrocytosis consumed less fat, and more niacin and ascorbic acid than anemics. Total iron intake was lower in anemic than the other groups, but without statistical significance due to the standard deviation of the data in a small number of anemic PSC (*n* = 16). TBI, unadjusted Hb, and adjusted Hb were not different between groups consuming or not multimicronutrients.

**Conclusions:**

The consumption of iron and iron status in children who live at high altitude is adequate, and that anemia could be due to other micronutrient deficiencies and/or that the adjustment of Hb by altitude is inappropriate.

**Supplementary Information:**

The online version contains supplementary material available at 10.1186/s40795-024-00837-x.

## Background

According to the last global statistics, by 2013, almost 27% (1,930 million people) worldwide was diagnosed as anemic [1]. From these, infants, children, and pregnant women constitute the most vulnerable groups [2]. Approximately 600 million pre-school and school-age children (24 months to 12 years) are diagnosed as anemic [2]. Worldwide, anemia prevalence in children under 5 years was reduced since 1990 to 2010, but then the rates were maintained stagnated [3].

According to the World Health Organization (WHO), 50% of anemia cases globally are attributable to iron deficiency (ID) [2, 5, 6]. Although, in recent studies the proportion of anemia associated with ID seems to be lower than the previously assumed 50% in countries with low, medium, or high Human Development Index ranking [7]. In Gambia, nearly 40% [8], in Azerbaijani Preschool Children (PSC), 27.0% [9] and in PSC from the Peruvian highlands, 21.97% [10] of anemia was attributable to ID.

One of the causes of ID and Iron Deficiency Anemia (IDA) is a low iron dietary intake. For such reason, most of intervention programs to reduce anemia is based on iron supplementation and/or iron food fortification [11]. The high prevalence of anemia contrasts with the fact that iron is one of the four most abundant metals in earth’s crust, and it is found in many of the nutrients consumed by humans [12].

In Peru, since 2004, a law N° 2831 enacted by government has required the fortification of foods for vulnerable populations such as infants under 36 months of age [13]. The fortification of foods with multiple micronutrients powders (MMPs) containing iron, decreases IDA and ID in pre-school and school-age children [3]. However, the effectiveness of this intervention in non-anemic children is not clear, and there is a debate about if children receiving iron in excess become adversely affected [14]. The risk of excessive intakes can be reduced by assessing baseline information on dietary intakes and voluntary use of supplements and continuously monitoring program coverage [15].

This high prevalence of anemia at high altitude (HA) seems to be due to the fact that in populations living over 1000 m altitude, WHO recommends an adjustment of hemoglobin value by altitude [16]. This correction increases prevalence of anemia as altitude increases, particularly over 3000 m above sea level [17]. This adjustment of Hb for altitude has been questioned by several authors [17–21].

After adjusting Hb for altitude, the prevalence of anemia in Bolivian children was 45.3%, whereas total body iron (TBI) measurements indicated that only 11.8% had tissue iron deficiency severe enough to produce anemia (TBI< -4 mg/kg) [17]. These differences in the estimated prevalence of IDA at HA has been suggested to be caused by an inappropriate altitude correction of the hemoglobin concentration. It is also possible that causes of anemia are others besides ID. For this, it is needed to know the iron dietary intake.

The region of Puno, in the Peruvian Southern Andes, is characterized by a high prevalence of anemia in children aged 6–59 months, and it is considered one of the highest values in the world [22]. In children 6–24 months of age residing at 3800 m in Puno, prevalence of anemia was 11.3% if Hb was not corrected by altitude, and increased to 94.7% after Hb correction [23]. This broad range of estimated prevalence complicates policy decisions, which are generally related to specific levels of disease prevalence.

The present study was designed to determine the dietary iron intake in preschoolers (PSC) adhering or not to iron supplementation in MMPs and its relationship with total body iron (TBI) content, and different categories of hemoglobin in a region of the highlands of Peru.

## Materials and methods

### Study design and participants

Cross-sectional study performed in the 13 provinces of the Region Puno, in southeastern Peru. This region is characterized because people live in zones with an average of 3800 m above sea level. The population size is 40,162 children, expected ratio: 50%, confidence level: 95.0% and design effect: 1.0. The distribution was proportional to the size of the strata. Sample size of the study was 338 children aged 6–59 months (173 were females and 165 were males) from urban (*n* = 223), urban marginal (*n* = 27) and rural (*n* = 88) places (Supplementary Table N°1) recruited throughout 2019. The inclusion criteria were children of 6 to 59 months-old, natives of the place of study. On the other hand, children vaccinated in the day of the study, children with acute diseases (with or without medication) including children with the presence of parasites evaluated by serial parasitological examination or children with chronic diseases were excluded (Supplementary Figure N°1).

### Biological samples

In each child, hemoglobin was measured in whole blood and biochemical markers of iron status and inflammation were measured in serum. Serum ferritin (SF, ng/mL), Soluble transferrin receptor (sTfR, ug/mL), Interleukin 6 (IL-6, pg/mL) levels were measured with ELISA kits (DRG International, INC, USA) according to manufacturer’s instruction.

Hemoglobin was measured using an automatized method, CELL-DYN Ruby®. Hemoglobin concentration was adjusted for altitude as suggested by the WHO, and called “altitude corrected Hb” [16], or it was maintained without adjustment called “uncorrected Hb”. Anemia was defined when Hb (corrected or uncorrected) is below 11 g/dl. For the calculation of the altitude corrected Hb the following formula is applied [16]:

Adjusted level = observed Hb level - altitude correction factor.

Altitude correction factor = 0.022 * ((altitude*altitude) − 0.032 (altitude).

Altitude= [(Altitude in meters) / 1 000] * 3.3.

Total Body Iron content (mg/Kg) was calculated using serum ferritin and sTfR values as previously reported [24] as follows:

$$ TBI\left(\frac{mg}{Kg}\right)=\frac{-[\text{log}\left(\frac{sTfR*1000}{sf}\right)-2.8229]}{0.1207}$$

ID was defined when TBI was < 0 mg/Kg >-4 mg/Kg. IDA was defined when TBI was < -4 mg/Kg [24]. It is known that inflammation may overestimate iron stores and misclassify an individual as iron sufficient. To avoid this, we have used the TBI calculation because is not affected by increased levels of IL-6 [10]. We have also calculated TBI in children with and without high IL6 levels. High IL-6 levels were defined when > 70 pg/ml [25].

### Dietary intake: 24-Hour Dietary Recall Method

Dietary intakes were assessed by a 24-hour dietary recalls (including dietary supplements) [26, 27], using NutriCap Software. The 24-hour reminder technique to caregivers of children aged 6 to 59 months allows to know the foods consumed by children for 24 h (day before), and then determine the amount of nutrients in the diet based in the Peruvian Food Composition Table [28]. The consumption of breast milk or formula was also considered.

Based on the 24-hour recall method, energy intake (Kilocalories), water, proteins, carbohydrates, fats, fiber, ash, vitamin A, ascorbic acid (AsA), folates, calcium, phosphorus, zinc, iron in vegetable-based, animal iron, and total iron intakes were calculated. The dietary recalls were conducted by trained and certified dietary interviewers at the School of Nutrition from the National University of Altiplano, Puno, and the participants were provided with a food model booklet and measurement aids for the diet recall interviews.

### Dietary iron intake

Estimated dietary intakes and their contents figuring in The Peruvian Food Composition Tables are used to calculate dietary intakes of iron [29]. Contribution of dietary iron is differentiated between intake of heme iron and non-heme iron. The first is calculated under the assumption that 40% of the iron of the meats, fish and poultry is in the heme form, and the difference between the total and the heme iron is obtained the non-heme iron [30, 31]. Non-heme iron is found mainly in plant-based foods, and in the remaining 60% of iron in animal products.

### Iron supplementation in multimicronutrient powder (MMP)

Considering that the Ministry of Health of Peru mandates the supplementation with iron in children 6–59 months, we have included data of children consuming at least 75% of the sachets supplied during the last month. Supplementation is done with sachets of MMPs Piramal healthcare manufactured by Piramal Enterprises Limited. Maharasha, India [32].

### Supplementation adherence

According to the Ministry of Health, adherence to the MMPs is considered adequate when ≥ 75% of the indicated dose is consumed [33]. This means that 23 or more sachets of MMPs are consumed in the last month. The percentage of adherence to supplementation was obtained through the survey of mothers or caregivers of the evaluated children.

### Ethical considerations

The study was approved by the Universidad Nacional del Altiplano (Puno, Peru), with code No. 3680-2017-R-UNA. All ethical considerations are contemplated, safeguarding the integrity of the children, and maintained data under confidentiality. Recruitment of children occurred after parents accepted voluntarily to participate. Children were recruited during visit for normal care after birth at the Health Facilities of the Ministry of Health (MoH) in Puno Region. Universidad Nacional del Altiplano shared the data base with Universidad Peruana Cayetano Heredia. Likewise, the project was registered as SIDISI: 104,908. Approval of the analysis of data was performed by the Institutional Review Board at the Universidad Peruana Cayetano Heredia (Code N°464-20-19).

### Statistical analysis

Data are presented as mean ± standard error of the mean (SEM). The STATA v17.0 (StataCorp, College Station TX) was used for analysis. The assumptions of normality and homogeneity of variances were evaluated. Differences between groups means were assessed with ANOVA test and Bonferroni as a post hoc test. Macro and micronutrients were assessed in groups defined as normal (Hb 11-14.5 g/dl), anemic (Hb < 11 g/dl) and erythrocytosis (Hb > 14.5 g/dl). Data were also assessed as groups depending of TBI values as < 0 mg/Kg, 0–5 mg/Kg and > 5 mg/Kg. TBI < 0 mg/Kg includes PSC with ID and IDA. Also, data were assessed according to adherence or not to MMPs. The coefficient of determination (R^2^) and the Pearson “r” correlation are used to study correlation between two quantitative variables. Multivariate linear regression analysis was applied to evaluate the association between TBI, ascorbic acid intake (mg/day), total iron intake (mg/day), and adherence to supplementation. The adjustment covariates were sex, age, and the altitude of residence. A statistical test is considered significant when *p* < 0.05.

## Results

### Prevalence of corrected and uncorrected anemia, and prevalence of ID and IDA

**Fig. 1:**
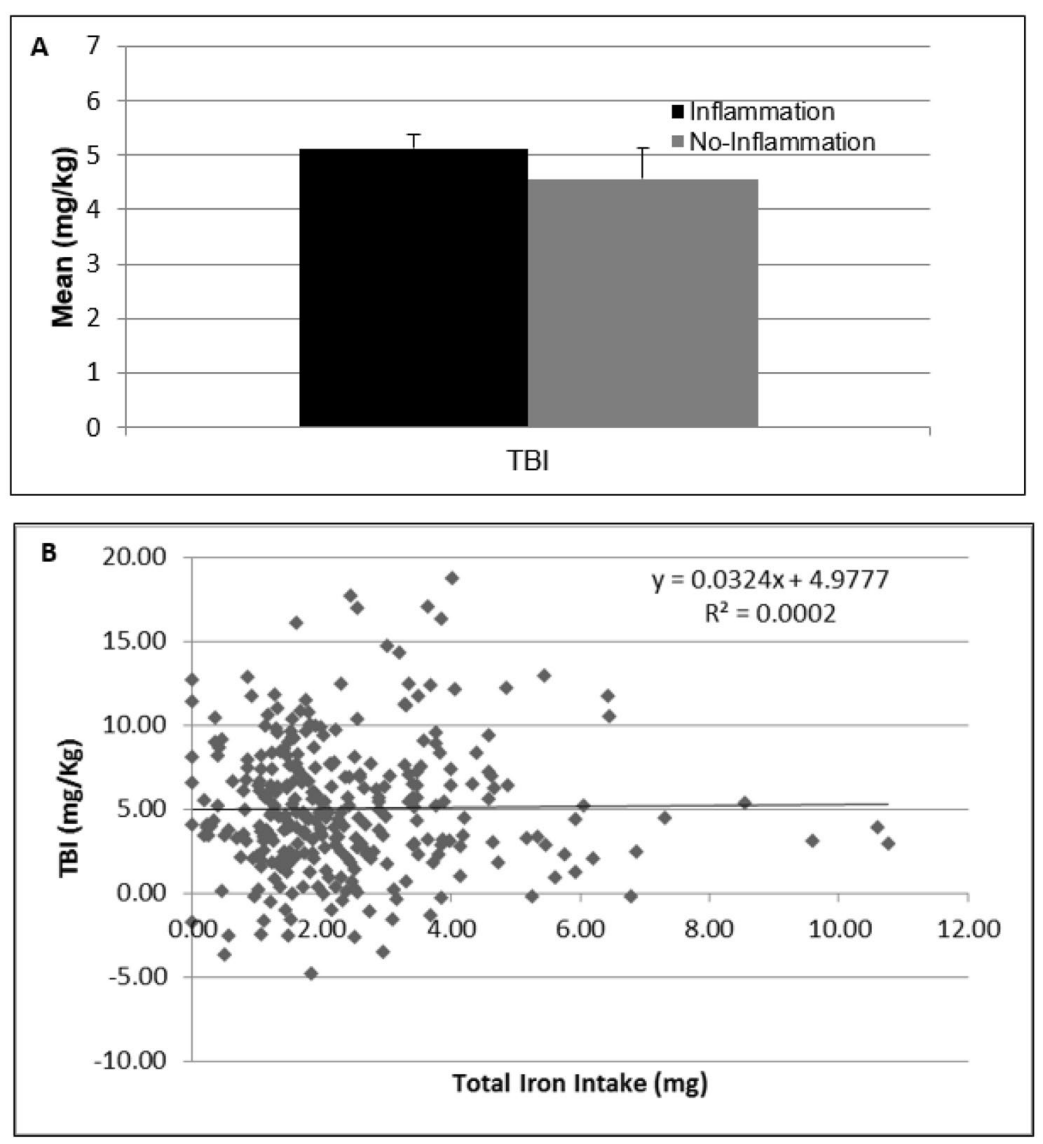
(**A**) Mean of TBI (mg/Kg). (Black chart) children with inflammation (IL6 > 70 pg/mL) and (Lead chart) without inflammation (IL6 ≤ 70 pg/mL). Bars are SEM. (**B**) Correlation between Total Iron Intake (mg) and TBI (mg/Kg) in Puno infants and preschoolers

With uncorrected Hb, the prevalence of anemia in PSC from Puno was 4.7% whereas after Hb correction for altitude, the prevalence of anemia raised-up to 65.6% (*p* < 0.001). Likewise, the correction of hemoglobin significantly reduced the proportion of children with erythrocytosis (Hb > 14.5 g / dL) from 20.35 to 0.30% (*p* < 0.001). TBI measurements showed that 7.44% has ID (TBI < 0 – ≥-4 mg/Kg) and 0.32% had IDA (TBI < -4 mg/Kg). TBI values were similar in PSC grouped as having or not inflammation (Fig. 1A).

### Correlation of macronutrient and energy intake with total iron intake

In Table 1A is shown the bivariate regression analysis between macronutrients (Independent variables) and total iron intake per day (Dependent variable) in PSC from Puno. There is a positive correlation between the consumption per day of calories, protein, carbohydrate or fat with the total iron intake (mg/day) (*p* < 0.01). This means that PSC consuming high macronutrients are also consuming high dietary iron, whereas, those PSC consuming low amounts of macronutrients are also consuming low amounts of iron. Better correlations were observed with non-heme iron (Table 1B) than with heme iron (Table 1C).

**Table 1:** Association between energy and macronutrient intake with (A) Total iron intake (mg/day), (B) Non-heme iron intake and (C) Heme iron Intake in children aged 6–59 months from Puno, Peru

**A. Total iron intake (mg/day)**	**Equation**	**R** ^**2**^	**R Pearson**	***p*** **-value**
Energy (Kcal/day)	y = 0.0068x + 1.8286	0.39	0.62	< 0.001
Protein (g/day)	y = 0.1421x + 2.5851	0.34	0.58	< 0.001
Carbohydrate(g/day)	y = 0.0345x + 2.4889	0.35	0.6	< 0.001
Fat (g/day)	y = 0.0971x + 5.5124	0.1	0.32	< 0.01
**B. Non-heme iron intake (mg/day)**	**Equation**	**R** ^**2**^	**R Pearson**	***p*** **-value**
Energy (Kcal/day)	y = 0.0061x + 1.5124	0.4	0.63	< 0.001
Protein (g/day)	y = 0.1240x + 2.3256	0.31	0.56	< 0.001
Carbohydrate(g/day)	y = 0.0334x + 1.7690	0.43	0.66	< 0.001
Fat (g/day)	y = 0.0745x + 5.0631	0.08	0.28	< 0.01
**C. Heme iron intake (mg/day)**	**Equation**	**R** ^**2**^	**R Pearson**	***p*** **-value**
Energy (Kcal/day)	y = 0.00061x + 0.4525	0.09	0.3	< 0.001
Protein (g/day)	y = 0.0233x + 0.1729	0.23	0.48	< 0.001
Carbohydrate(g/day)	y = 0.00146x + 0.7445	0.02	0.14	< 0.05
Fat (g/day)	y = 0.02117x + 0.5537	0.13	0.28	< 0.01

Data are obtained from 338 children aged 6–59 months from Puno, Peru. R^2^: Coefficient of determination. Data does not include MNPs supplementation

Equations of linear regression analysis of the consumption of energy, protein, carbohydrates, fats (independent variable) with **(A)** Total iron and its categories of **(B)** Non-heminic and **(C)** heminic iron (dependent variable)

### Dietary composition between hemoglobin status, TBI and adherence to supplementation

In Table 2 is observed the mean amount of consumption for different macronutrients and micronutrients in regard of hemoglobin status without the use of the Hb correction factor for definition of anemia (Hb < 11 g/dl) (*n* = 16), normal Hb values (Hb = 11-14.5 g/dl) (*n* = 238) and erythrocytosis (Hb > 14.5 g/dl) (*n* = 84). PSC having normal Hb levels consumed more protein, beta carotene, niacin, AsA and sodium than anemic PSC. PSC with erythrocytosis consumed less fat, and more niacin and AsA than anemic children.

**Table 2:** Dietary composition of children aged 6 to 59 months in the Puno, Peru, according to anemia, normal and erythrocytosis based on uncorrected Hb

Nutrient	Anemia	Normal	Erythrocytosis
Energy (Kcal)	723.49 ± 56.23	824.51 ± 20.27	782.19 ± 31.04
Water	404.79 ± 46.88	484.23 ± 11.55	447.96 ± 18.46
Protein (g)	28.88 ± 1.90	33.65 ± 0.84**	33.15 ± 1.62
Fat (g)	20.27 ± 1.62	19.61 ± 0.75	15.30 ± 0.95*
Total Carbohydrate (g)	118.53 ± 11.57	141.46 ± 3.76	140.79 ± 6.41
Bioavailable Carbohydrate (g)	65.23 ± 7.35	74.38 ± 2.02	72.58 ± 3.68
Fiber (g)	7.93 ± 0.79	10.02 ± 0.32	10.21 ± 0.63
Ash (g)	4.93 ± 0.43	5.68 ± 0.16	5.59 ± 0.26
Calcium (mg)	309.28 ± 51.64	293.84 ± 14.56	274.47 ± 22.89
Phosphorous (mg)	480.17 ± 38.95	533.60 ± 15.17	537.02 ± 26.21
Zinc (mg)	4.38 ± 0.68	4.62 ± 0.16	4.84 ± 0.38
Heme iron (mg)	4.19 ± 1.47	4.02 ± 0.34	5.34 ± 0.7
Non-heme iron (mg)	6.07 ± 0.60	6.52 ± 0.19	6.30 ± 0.32
Total iron (mg)	10.26 ± 1.62	10.56 ± 0.37	11.65 ± 0.75
Beta Carotene (ug)	598.72 ± 122.34	939.89 ± 52.12**	794.92 ± 83.26
Vitamin A (ug)	578.10 ± 221.33	500.34 ± 37.70	615.33 ± 111.47
Thiamin (mg)	0.45 ± 0.06	0.56 ± 0.02	0.57 ± 0.04
Riboflavin (mg)	0.90 ± 0.11	0.91 ± 0.04	0.89 ± 0.06
Niacin (mg)	5.12 ± 0.64	6.98 ± 0.24*	7.09 ± 0.63*
Ascorbic acid (mg)	45.25 ± 10.85	55.15 ± 3.71**	63.67 ± 6.89*
Sodium (mg)	25.50 ± 5.47	45.51 ± 5.17*	27.76 ± 2.45
Potassium (mg)	4461.38 ± 858.36	4620.06 ± 398.99	3383.03 ± 368.75
Folate (ug)	268.94 ± 55.50	254.13 ± 10.32	274.14 ± 16.60
**TBI (mg/Kg)**	4.26 ± 1.30	5.56 ± 0.27	3.74 ± 0.34^#^
**IL-6 (pg/ml)**	48.39 ± 6.84	45.08 ± 1.58	47.1 ± 2.5

Anemia was defined as Hb < 11 g/dl. Erythrocytosis was defined if Hb > 14.5 g/dl

Data are mean ± SEM.

ANOVA Test: *p < 0.01; **p < 0.05 with respect to the group with anemia; # *p* < 0.05 with respect to the normal group

Data includes MNPs supplementation

Total iron intake was lower in anemic than in non-anemic PSC or PSC with erythrocytosis. However, there is not statistical significance due to the deviation of the data in a small number of anemics in the group (*n* = 16). Serum IL6 levels were not different between groups (*p* > 0.05). Although mean TBI was lower in anemic than in normal Hb PSC, no statistical difference was observed.

Table 3 shows that the amount consumed of macronutrients and micronutrients is similar between the three categories of TBI [TBI < 0 mg/Kg (*n* = 24); 0–5 mg/kg (*n* = 170) and > 5 mg/Kg (*n* = 144)], except with intake of AsA and sodium. The group with highest TBI values was associated with high AsA and sodium intake, 63.85 and 47.88 mg, respectively. Serum IL6 levels were not different between groups. PSC with TBI 0–5 mg/Kg and > 5 mg/Kg consumed more non-heme iron than heme iron compared with the group of TBI < 0 mg/kg.

**Table 3:** Dietary composition in children aged 6 to 59 months in the Puno region according to iron status, stratified in three categories of total body iron (TBI): TBI less than 0 mg/kg; between 0–5 mg/kg and more than 5 mg/kg

Nutrient	TBI < 0	TBI 0–5	TBI > 5
Energy (Kcal)	802.86 ± 73.98	814.90 ± 23.21	807.07 ± 25.11
Water	453.49 ± 34.52	464.97 ± 13.00	483.21 ± 15.86
Protein (g)	33.07 ± 2.99	33.24 ± 1.05	33.46 ± 1.05
Fat (g)	21.32 ± 2.74	18.08 ± 0.76	19.04 ± 1.01
Total Carbohydrate (g)	132.92 ± 12.26	142.73 ± 4.58	138.49 ± 4.62
Bioavailable Carbohydrate (g)	67.46 ± 5.30	74.69 ± 2.43	73.39 ± 2.70
Fiber (g)	8.83 ± 0.91	10.18 ± 0.39	9.89 ± 0.42
Ash (g)	5.34 ± 0.47	5.62 ± 0.18	5.68 ± 0.20
Calcium (mg)	307.99 ± 50.32	274.67 ± 15.35	306.14 ± 20.06
Phosphorous (mg)	529.83 ± 53.48	529.83 ± 17.74	534.58 ± 19.25
Zinc (mg)	4.95 ± 0.63	4.61 ± 0.21	4.66 ± 0.23
Heme iron (mg)	5.06 ± 1.27	4.22 ± 0.42	4.30 ± 0.46
Non-heme iron (mg)	5.92 ± 0.53	6.67 ± 0.23^#^	6.30 ± 0.23^#^
Total iron (mg)	10.98 ± 1.48	10.88 ± 0.46	10.61 ± 0.50
Beta Carotene (ug)	655.69 ± 114.46	833.85 ± 57.42	1002.06 ± 71.46
Vitamin A (ug)	536.29 ± 158.61	517.81 ± 55.41	539.86 ± 55.06
Thiamin (mg)	0.50 ± 0.05	0.59 ± 0.02	0.52 ± 0.02
Riboflavin (mg)	0.89 ± 0.12	0.96 ± 0.04	0.84 ± 0.03
Niacin (mg)	6.58 ± 0.52	6.92 ± 0.34	6.96 ± 0.33
Ascorbic acid (mg)	49.74 ± 8.15	51.21 ± 3.09	63.85 ± 6.26**
Sodium (mg)	30.24 ± 3.95	36.12 ± 3.15	47.88 ± 8.21**
Potassium (mg)	4415.29 ± 949.35	3922.24 ± 281.34	4813.19 ± 626.34
Folate (ug)	257.63 ± 35.60	248.21 ± 10.74	272.19 ± 15.17
**TBI (mg/Kg)**	-1.52 ± 0.26	2.82 ± 0.11	8.38 ± 0.24
**IL-6 (pg/ml)**	43.7 ± 4.62	46.83 ± 1.93	44.62 ± 61.96

Data are mean ± SEM.

ANOVA Test: *p < 0.01; **p < 0.05 with respect to the group with TBI < 0.

T-student Test: #*p* < 0.0001 with respect heme iron into the same TBI group

Data includes MNPs supplementation

The percentage of PSC with adherence to MMPs supplementation is 27.55% (*n* = 91) compared with around 72% (*n* = 239) of PSC without adherence to supplementation (239) (*p* < 0.001). In addition, the consumption of macro and micronutrients was greater and significant in PSC without adherence to supplementation. Adherence to supplementation was also associated with lower age in PSC. TBI, Hb, and altitude corrected Hb were not different between groups consuming or not supplements of MMPs (Table 4).

**Table 4:** Dietary composition in children aged 6 to 59 months in the Puno region with or without adherence to supplementation and nutrients (macro and micronutrients)

Nutrient	Total	With adherence to supplement	Without adherence to supplement
Energy (Kcal)	812.97 ± 16.95	730.68 ± 27.64	844 ± 20.58*
Water	473.12 ± 9.84	440.91 ± 15.54	485.41 ± 12.16**
Protein (g)	33.42 ± 0.73	30.47 ± 1.12	34.54 ± 0.91*
Fat (g)	18.81 ± 0.62	16.57 ± 1.12	19.66 ± 0.73**
Total Carbohydrate (g)	140.56 ± 3.21	126.99 ± 5.29	145.72 ± 3.89*
Bioavailable Carbohydrate (g)	73.48 ± 1.74	69.3 ± 2.9	75.1 ± 2.11
Fiber (g)	10.02 ± 0.27	9.24 ± 0.51	10.32 ± 0.33
Ash (g)	5.66 ± 0.13	5.22 ± 0.22	5.82 ± 0.16**
Calcium (mg)	292.68 ± 12.29	278.61 ± 20.55	298.04 ± 15.08
Phosphorous (mg)	533.67 ± 12.81	489.42 ± 19.39	550.52 ± 15.97*
Zinc (mg)	4.70 ± 0.15	8.05 ± 0.15	3.43 ± 0.12*
Heme iron (mg)	4.40 ± 0.31	13.37 ± 0.06	0.99 ± 0.04*
Non-heme iron (mg)	6.48 ± 0.16	5.82 ± 0.25	6.74 ± 0.19*
Total iron (mg)	10.89 ± 0.33	19.20 ± 0.28	7.73 ± 0.22*
Beta Carotene (ug)	898.89 ± 43.54	906.42 ± 62.82	896.02 ± 55.22
Vitamin A (ug)	535.24 ± 38.83	793.10 ± 75.04	437.06 ± 43.83*
Thiamin (mg)	0.55 ± 0.01	0.49 ± 0.02	0.58 ± 0.02*
Riboflavin (mg)	0.91 ± 0.03	0.85 ± 0.04	0.93 ± 0.03
Niacin (mg)	6.94 ± 0.23	6.39 ± 0.33	7.15 ± 0.29
Ascorbic acid (mg)	56.86 ± 3.22	68.76 ± 4.28	52.34 ± 4.10*
Sodium (mg)	40.93 ± 3.90	30.31 ± 2.08	44.97 ± 5.29*
Potassium (mg)	4402.46 ± 314.25	5566.94 ± 960.47	3925.19 ± 228.46
Folate (ug)	261.54 ± 8.92	363.70 ± 16.92	222.65 ± 9.35*
**Age (months)**	34.79 ± 0.77	28.59 ± 1.41	37.15 ± 0.87*
**TBI (mg/Kg)**	5.08 ± 0.21	4.92 ± 0.43	5.15 ± 0.25
**Hemoglobin (g/dl)**	13.48 ± 0.07	13.49 ± 0.16	13.47 ± 0.08
**Corrected hemoglobin (g/dl)**	10.41 ± 0.08	10.34 ± 0.16	10.43 ± 0.09

Data are mean ± SEM.

T-Student Test: *p < 0.01; **p < 0.05 respect to the group with adherence to MNPs.

### Correlation between iron intake, ascorbic acid intake and adherence to program with total body iron

A linear correlation analysis showed that there is no correlation between amount of total iron consumption and TBI levels (Fig. 1B). After adjusting for covariates, the same result was observed, the intake of iron does not change TBI content (*p* = 0.8); while the consumption of AsA increases TBI content (*p* = 0.007). Adherence to MMPs supplementation was not related to TBI (*p* = 0.746) (Table 5).

**Table 5:** Multiple linear regression to determine association of ascorbic acid intake (mg/day), total iron intake (mg/day), adherence to supplementation with Total Body Iron (TBI).

TBI (mg/Kg)	Coefficient ± SE	*p*-value	95% CI
Ascorbic acid intake (mg/day)	0.01061 ± 0.004	0.007	0.00289–0.0183
Total iron intake (mg/day)	-0.0183 ± 0.072	0.800	-0.16029-0.123
Adherence to supplementation	0.3231 ± 0.998	0.746	-1.640-2.286

Model adjusted by sex, age (months) and altitude of residence (meters above sea level)

## Discussion

The present study was designed to analyze the dietary composition between categories of hemoglobin, TBI, and adherence to MMPs of PSC living at the highlands in the Southern Peruvian Andes. In the present study, 4.7% of PSC were diagnosed as anemic but when Hb was adjusted for altitude the prevalence of anemia increased to 65.6%.

One important finding is that PSC consuming low macronutrients consumes also low iron amounts in the diet. Therefore, PSC with anemia has fewer intakes of protein, fiber, beta carotene, niacin, AsA and sodium. These differences disappear when Hb is corrected by altitude. This happens because all PSC with erythrocytosis except one are re-classified as normal Hb and many PSC with normal uncorrected Hb are diagnosed as anemic after Hb correction for altitude. Several authors suggest that Hb should not be corrected by altitude [17, 19, 34, 35]. The arguments against the correction of Hb for altitude include the fact that the increase in Hb due to altitude is not universal and will depend on ethnicity and length of multigenerational seniority [36].

Population with longer data living at HA as native highlanders originating from the Tibetan and the Ethiopian plateaus present with a normal or only mildly elevated hemoglobin concentration [37]. People from ethnia Han in the Tibet with only 70 years living at HA have elevated levels of Hb compared with Tibetans living there for almost 25,000 years.

In addition, in Peru, the proportion of anemia attributable to ID was 22% of cases of anemia in children aged 6–59 months, and the proportion of anemia attributable to inflammation (27.8%). As other causes have not been identified is plausible to think that most of the cases of anemia at HA not due to ID or inflammation is due to the adjustment of Hb for altitude [10]. Moreover, populations living at HA have normal or higher iron status than those at low altitudes [38].

In Tibetan subjects with normal iron status and without deficiency of other vitamins, without hemoglobinopathies, anemia rates were very low but increase notably after Hb adjustment for altitude. Tibetan men had an apparent anemia (hemoglobin < 13 g/dL) prevalence of 1.4% (one male) and women had no anemia (hemoglobin < 12 g/dL). The WHO-recommended altitude adjustment, established using data on Andean highlanders, raised the prevalence of apparent anemia among Tibetan men to 77.8% (< 16.5 g/dL) and 86.5% (< 15.4 g/dL) among women [19].

According to our results, mean iron intake in PSC from Puno, Peru, is 10.78 ± 0.33 mg/day. This value is higher than the reported for Mexican children aged 12–50 months-old, residing also at high altitude. Mexican PSC has a mean iron intake of 6.2 ± 4.4 mg/day [39]. However, despite of this difference in iron intake, the prevalence of anemia after Hb adjustment is significantly higher in Puno (65.6%) than in the Mexican children (22.5%) [40]. Puno has in average more altitude (3800 m) than Mexico DF (2250 m).

The finding that only 7.44% of PSC has ID (TBI < 0 – ≥-4 mg/Kg) and 0.32% had IDA (TBI <-4 mg/Kg) suggest that high prevalence of anemia after Hb correction (65.6%) is not real.

Although mean total iron intake was lower in anemic PSC from Puno, the absence of significance seems to be due to the high deviation and to the low number of anemic PSC. This means that anemic PSC includes those with normal iron intake and others with low iron intake. A lack of difference in iron intake between adolescent with anemia and in those without anemia has been also reported [41]. This is in accordance to the fact that anemia has different causes and not only low iron intake [10]. In fact, children with IDA had significantly lower intakes of energy, protein, fat and various micronutrients, compared to those with normal iron status [42].

It is interesting to find that children with high TBI content has also more intake of AsA than children with ID and IDA (TBI < 0 mg/Kg). Several studies have demonstrated that AsA is an important enhancer of iron absorption not only supporting with an acid environment to the duodenum [43–46] but also regulating the of hypoxia inducible factor (HIF) which sense oxygen availability and iron homeostasis [47].

Low intake of niacin and beta carotene in PSC from Puno was associated with anemia as observed in other studies [48, 49].

Our study showed that only AsA was reduced in the anemic group. Supplementation with MMPs includes ascorbic acid (30 mg/sachet) but according to our analysis, this value seems to be insufficient to increase Hb concentration. Other authors recommend, 100–200 mg/day in other study [50]. These values are greater than that reported as average intake in our study.

The absorption of iron by the duodenum is essential to maintain its balance in the body, since, unlike most other essential nutrients; iron is not excreted in humans, this allows iron homeostasis. Also, this system has been developed to avoid iron overload that may have adverse effects in tissues [51].

A dietary intake contains heme and non-heme iron [52]. Heme iron has high bioavailability (15–35%) respect to non-heme iron (1–20%) [53]. Heme iron is composed of ferrous cation (Fe2+) and it is suggested to be absorbed as an intact metalloprotein via heme carrier protein 1 (HCP-1). In the enterocyte, ferrous iron is released from heme via heme oxygenase [54, 55]. Non-heme iron requires the conversion of the ferric (Fe3+) to ferrous cation which occurs in acid environments and depends on the divalent metal transporter 1 (DMT 1) to transport iron inside the enterocyte [56]. Thereafter, iron requires ferroportin to be exported to the systemic circulation. Ferroportin is down regulated by hepcidin [57].

Although fine regulation of iron absorption occurs to avoid iron overload in normal children, more recently, an alert has emerged from studies on gut microbiota [14, 58]. If a child receives an excess of iron it affects the gut microbiota by increasing the enteropathogenic count [59]. This in turn may result in systemic inflammation which could produce an anemic state [60].

Different studies suggest that modest response to iron interventions seems to be due mainly to a low adherence to the supplementation [61, 62]. However, none of these studies have reported data on dietary iron intake in children and how adherence to iron supplementation affects iron status. Then, it is necessary to know through nutritional surveys the daily dietary iron intake. Previous study showed that, both heminic and non-heminic iron were positively associated with serum ferritin [63].

Therefore, a contribution of this research is the evaluation of the nutritional composition, in particular the consumption of iron. An adequate consumption of iron results in normal TBI and, therefore, individuals are considered full of iron [63]. Likewise, the evaluation of the enhancing [64] and inhibitors [65] compounds of iron absorption will allow to know what is the best way to handle the type of diet used to fight against anemia.

In a population where iron is enough as observed in the present study in PSC from Puno, the supplementation with MMPs will not increase further TBI.

In populations where iron is deficient, home fortification with MMPs, compared with no intervention or placebo, reduced the risk of anemia in infants and increased hemoglobin concentrations and presented higher iron status [11]. The results of a meta-analysis in Latin America, evidenced that nutritional intervention reduced the prevalence of anemia from 45 to 25% [6].

The main strength of the study is the inclusion of nutritional and hematological evaluation which allow to know the real iron status of these children.

This is a cross-sectional study, which does not establish a causality between the variables studied. Likewise, the methodology of the 24-hour recall was only applied once; however, this technique has been widely used for nutritional evaluation, both by researchers and by international institutions and organizations.

## Conclusions

A major conclusion is that in children and preschoolers from Puno, iron supplementation is not necessary. Consumption of macro and micronutrients in the Southern Andes is adequate, contradicting the premises of the policies carried out in the majority of countries with high proportion of the population living at the highlands, where the prevalence of anemia seems to be high due to the adjustment of Hb by altitude or that causes of anemia are other different to iron deficiency. Likewise, the importance of measuring nutrients and antinutrients to ensure adequate absorption of iron should be emphasized. The strong association evidenced between the high consumption of ascorbic acid and the increase in body iron content, demonstrating that a diet should be governed by not exclusively macronutrients and micronutrients, but also with an adequate ratio between iron and AsA consumption.

### Electronic supplementary material

Below is the link to the electronic supplementary material.

Supplementary Material 1

Supplementary Material 2

Supplementary Material 3

## Data Availability

The datasets used and/or analysed during the current study are available from the corresponding author on reasonable request.
