# Supplementary material for: Evaluation of dietary composition between hemoglobin categories, total body iron content and adherence to multi-micronutrients in preschooler residents of the highlands of Puno, Peru
Source: BMC Nutr. 2024 Feb 12;10:28. doi: 10.1186/s40795-024-00837-x (PMC10860272; doi:10.1186/s40795-024-00837-x)
Supplement: Supplementary file 1 — Supplementary Material 1 [file 40795_2024_837_MOESM1_ESM.docx]

**Supplementary Table 1.** Dietary composition in children aged 6 to 59 months in the Puno region according to residential zone, stratified in three categories: urban, rural and urban marginal area.

| **Nutrient** | **Urban area** | **Rural area** | **Urban marginal area** |
| --- | --- | --- | --- |
| Energy (Kcal) | 808.53±20.70 | 817.70±35.0 | 810.08±59.7 |
| Water | 474.44±11.94 | 462.57±19.31 | 489.63±43.25 |
| Protein (g) | 33.39±0.90 | 33.26±0.51 | 33.60±2.38 |
| Fat (g) | 19.07±0.81 | 17.86±1.09 | 18.07±1.85 |
| Total Carbohydrate (g) | 139.02±3.79 | 143.31±6.91 | 140.15±11.96 |
| Bioavailable Carbohydrate (g) | 73.52±2.15 | 74.85±3.54 | 70.08±6.60 |
| Fiber (g) | 9.96±0.34 | 10.05±0.54 | 8.85±0.96 |
| Ash (g) | 5.60±0.16 | 5.68±0.27 | 5.75±0.46 |
| Calcium (mg) | 285.30±13.74 | 310.97±29.76 | 288.85±35.36 |
| Phosphorous (mg) | 529.10±15.79 | 544.37±26.81 | 529.84±40.61 |
| Zinc (mg) | 4.74±0.19 | 4.46±0.32 | 4.57±0.44 |
| Heme iron (mg) | 4.43±0.38 | 4.11±0.59 | 4.13±1.04 |
| Non-heme iron (mg) | 6.32±0.19 | 6.91±0.36 | 6.14±0.49 |
| Total iron (mg) | 10.75±0.41 | 11.036±0.69 | 10.27±1.13 |
| Beta Carotene (ug) | 852.49±48.02 | 907.15±87.53 | 971.16±162.72 |
| Vitamin A (ug) | 488.31±43.10 | 634.13±95.69 | 529.96±112.08 |
| Thiamin (mg) | 0.60±0.023 | 0.57±0.034 | 0.60±0.073 |
| Riboflavin (mg) | 0.92±0.040 | 0.88±0.053 | 1.02±0.095 |
| Niacin (mg) | 6.81±0.27 | 7.32±0.53 | 6.85±0.73 |
| Ascorbic acid (mg) | 55.40±3.28 | 56.65±6.13 | 50.81±6.85 |
| Sodium (mg) | 41.57±4.17 | 42.43±10.75 | 35.94±5.03 |
| Potassium (mg) | 4511.98±452.07 | 4209.299±391.69 | 3655.60±664.65 |
| Folate (ug) | 265.77±11.96 | 241.70±13.71 | 248.92±25.12 |
| Ferritin (ng/ml) | 22.60±2.11 | 19.36±1.23 | 24.36±2.58 |
| Hepcidin (ng/ml) | 24.62±1.34 | 24.08±1.80 | 23.96±3.52 |
| sRTf/log(ferritin)(ug/ml) | 3.25±1.84 | 3.34±0.25 | 3.48±0.61 |
| Erythropoietin (mIU/ml) | 23.16±1.19 | 20.93±0.97 | 20.74±1.02 |
| **IL-6 (pg/ml)** | **47.94±1.81** | **43.41±2.12** | **36.26±3.84** |
| **TBI (mg/Kg)** | **5.099±0.27** | **4.68±0.39** | **5.85±0.98** |

Data are mean ± SEM.
