# Supplementary material for: Evaluation of dietary composition between hemoglobin categories, total body iron content and adherence to multi-micronutrients in preschooler residents of the highlands of Puno, Peru
Source: BMC Nutr. 2024 Feb 12;10:28. doi: 10.1186/s40795-024-00837-x (PMC10860272; doi:10.1186/s40795-024-00837-x)
Supplement: Supplementary file 2 — Supplementary Material 2 [file 40795_2024_837_MOESM2_ESM.docx]

**Supplementary Table 2.** Dietary composition of children aged 6 to 59 months in the Puno Region according to hemoglobin (Hb) status with correction, stratified in three categories: corrected anemia, normal Hb and corrected erythrocytosis (Hb>14.5g/dL)^#^.

| **Nutrient** | **Corrected Anemia** | **Normal^#^** |
| --- | --- | --- |
| Energy (Kcal) | 812.28±21.48 | 817.66±26.26 |
| Water | 474.51±12.69 | 467.47±15.01 |
| Protein (g) | 32.77±0.88 | 34.96±1.30 |
| Fat (g) | 19.39±0.78 | 17.73±0.97 |
| Total Carbohydrate (g) | 139.44±4.03 | 143.07±5.09 |
| Bioavailable Carbohydrate (g) | 73.15±2.16 | 75.13±2.88 |
| Fiber (g) | 9.92±0.33 | 10.71±0.49 |
| Ash (g) | 5.49±0.16 | 5.96±0.23 |
| Calcium (mg) | 288.80±15.01 | 296.36±21.21 |
| Phosphorous (mg) | 522.66±15.25 | 559.60±23.25 |
| Zinc (mg) | 4.64±0.17 | 4.70±0.28 |
| Heme iron (mg) | 4.17±0.37 | 4.51±0.54 |
| Non-heme iron (mg) | 6.58±0.21 | 6.34±0.24 |
| Total iron (mg) | 10.71±0.40 | 10.85±0.57 |
| Beta Carotene (ug) | 899.52±55.53 | 910.40±69.12 |
| Vitamin A (ug) | 523.47±43.59 | 530.25±74.33 |
| Thiamin (mg) | 0.54±0.02 | 0.58±0.03 |
| Riboflavin (mg) | 0.90±0.03 | 0.92±0.05 |
| Niacin (mg) | 6.79±0.27 | 7.22±0.43 |
| Ascorbic acid (mg) | 54.78±3.97 | 60.33±5.33 |
| Sodium (mg) | 44.10±5.77 | 34.49±2.63 |
| Potassium (mg) | 4636.17±441.95 | 3840.60±308.55 |
| Folate (ug) | 212.68±9.51 | 230.77±15.55 |
| **TBI (mg/Kg)** | **4.34±0.33** | **5.40±0.29** |
| **IL-6 (pg/ml)** | **45.28±1.47** | **46.71±2.68** |

Anemia was defined as Hb<11 g/dl. # Corrected erythrocytosis was not included in the analysis because the number of subjects was 1.

Data are mean ±SEM. *p><0.05 with respect to the group with anemia.

Data includes MNPs supplementation.
