# Supplementary material for: Evaluation of dietary composition between hemoglobin categories, total body iron content and adherence to multi-micronutrients in preschooler residents of the highlands of Puno, Peru
Source: BMC Nutr. 2024 Feb 12;10:28. doi: 10.1186/s40795-024-00837-x (PMC10860272; doi:10.1186/s40795-024-00837-x)
Supplement: Supplementary file 3 — Supplementary Material 3 [file 40795_2024_837_MOESM3_ESM.docx]

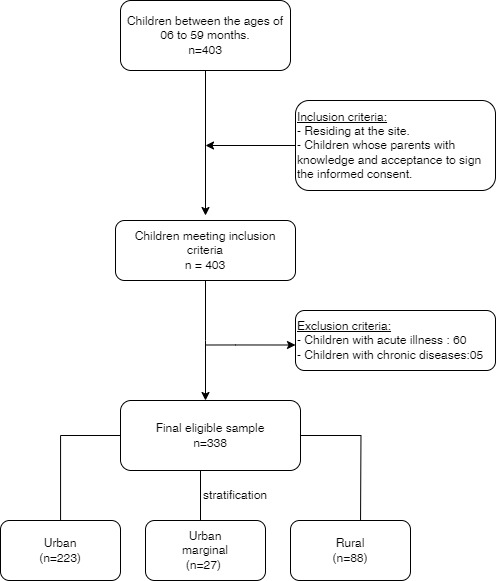


**Supplementary figure N°1.** Flow chart of the study participants, children aged 06 to 59 months residing in Puno, Peru.
